# A fortuitous find: a unique haplotype of *Ooencyrtus nezarae* Ishii (Encyrtidae: Encyrtinae) discovered in Florida

**DOI:** 10.3897/BDJ.8.e36440

**Published:** 2020-01-17

**Authors:** Nicholas C Goltz, Jessica Awad, Matthew R Moore, Elijah J Talamas

**Affiliations:** 1 University of Florida, Gainesville, United States of America University of Florida Gainesville United States of America; 2 Florida Department of Agriculture and Consumer Services, Division of Plant Industry, Gainesville, United States of America Florida Department of Agriculture and Consumer Services, Division of Plant Industry Gainesville United States of America

**Keywords:** *Megacopta cribraria*, *Paratelenomus saccharalis*, biological control, fortuitous biological control, species delimitation, DNA barcoding

## Abstract

The adventive arrival of biological control agents circumvents the regulatory process by introducing exotic species to control invasive pests and is generally followed by post hoc risk evaluation. The bean plataspid, *Megacopta cribraria* (Fabricius) (Hemiptera: Plataspidae), is an invasive pest of leguminous crops in the south-eastern United States that was eventually followed by two parasitoid wasps from its range in the eastern hemisphere, *Paratelenomus saccharalis* (Dodd) (Scelionidae) and *Ooencyrtus nezarae* Ishii (Encyrtidae). In North Central Florida, sentinel egg masses, intended to capture *Paratelenomus saccharalis*, instead yielded *Ooencyrtus nezarae*, which was previously known only from Alabama (Ademokoya et al. 2018). Two generations of *O. nezarae* were subsequently reared in the laboratory. COI sequences from the Florida population of *O. nezarae* differed by 1.3% from the Alabama population and the presence of a different haplotype suggests the possibility of a separate introduction. Laboratory parasitism rates, sex ratios, morphology, molecular diagnosis and implications for agriculture are discussed.

## Introduction

The term “fortuitous biological control” was coined by DeBach (1971), referring to the unintentional introduction of natural enemies of invasive species. Although the original essay is obscure, the phenomenon received further treatment in DeBach and Rosen (1991), who estimated that 43% of exotic beneficial arthropods in the United States arrived by accident. Their analyses focused on parasitoids of scale insects, reasoning that parasitoids, whose hosts are small and sessile on plants, are easily moved through international agricultural trade.

This phenomenon has received renewed attention in recent years, due in part to the high-profile case of the samurai wasp, *Trissolcus japonicus* (Ashmead) (Hymenoptera: Scelionidae) (Talamas et al. 2015, Milnes et al. 2016, Servick 2018, Stahl et al. 2018). Scientists spent years evaluating the feasibility of introducing this Asian wasp species for control of the brown marmorated stink bug, *Halyomorpha halys* Stål (Hemiptera: Pentatomidae), in North America. While host specificity experiments were underway, wild populations of *T. japonicus* were discovered in Maryland. Genetic analysis revealed that wild populations were unrelated to laboratory strains. This, consequently, eliminated the possibility of an escape from quarantine and suggested that the samurai wasp found its own way to the New World (Talamas et al. 2015).

Pentatomoid eggs, like the scale insects studied by DeBach and Rosen (1991), are small, sessile and easily transported on plant products. Thus, egg parasitoids have the potential to arrive adventively in new areas. In addition to *T. japonicus*, three other species of hymenopteran egg parasitoid of pentatomoids have unexpectedly emerged in the United States within the last five years: *T. hyalinipennis* Rajmohana & Narendran, a parasitoid of *Bagrada hilaris* (Burmeister) (Pentatomidae), was discovered in California (Ganjisaffar et al. 2018); *Paratelenomus saccharalis*, a specialist egg parasitoid of *Megacopta cribraria*, was detected in Georgia and Florida (Gardner et al. 2013, Medal et al. 2015); and *Ooencyrtus nezarae*, a generalist egg parasitoid, was reared from *M. cribraria* in Alabama in 2016 (Ademokoya et al. 2018).

We recently discovered wild *O. nezarae* in North Central Florida. The publication of COI barcodes from the Alabama population of *O. nezarae* facilitated the identification of the parasitoids found in Florida. We here present rearing data, including observations of parasitism and emergence rates, morphological description and COI barcoding results for this population of *O. nezarae*.

## Material and methods

### Collection and rearing

On June 19, 2018, a collection trip was undertaken to replenish the colony of *Paratelenomus saccharalis* at the Florida Department of Agriculture and Consumer services – Division of Plant Industry (FDACS-DPI) in Gainesville, Florida. A patch of kudzu, Pueraria montana var. lobata (Lour.) Merr. (Fabaceae) located in Alachua County, Florida (29.8055°N, 82.5301°W) was chosen as the collection site due to its ease of access and favorable conditions for *P. saccharalis* development. At the site, kudzu foliage bearing *M. cribraria* eggs was carefully cut and collected to be evaluated for parasitism at the lab.

Of these *M. cribraria* egg masses, 47 were identified as having an irregular, ash-grey colouration, a common sign of parasitism (Gardner et al. 2013). These egg masses were separated from the others and placed in a 40 cm x 30 cm x 30 cm plexiglass growth chamber supplied with honey-soaked strips of paper and water for the adult parasitoids to access *ad libitum*. The growth chamber was maintained at 25–27°C, 70–82% humidity and on a 16L:8D light cycle.

### Parasitism and emergence

Thirty *Ooencyrtus nezarae* adults were separated into three glass vials (V = 31 cm^3^ ; 10 parasitoids in each with an unknown sex ratio), each containing one *M. cribraria* egg mass and a honey-soaked strip of paper. Egg masses were obtained from captive colonies and stored in a freezer for 6 – 14 months before exposure. These vials were then sealed and maintained at 25 – 27°C, 60 – 78% humidity with a 16L:8D light cycle. The remaining parasitoids in the growth chamber were provided with four previously frozen, lab-reared *M. cribraria* egg masses and were maintained under the conditions described above.

Adult *O. nezarae* emergence in the glass vials was first observed 14 days later and continued for several more days. Once it was determined that adult parasitoids were unlikely to continue emerging, the 122 adults that emerged were individually placed in separate gelatine capsules and sorted by sex, resulting in 36 males and 86 females. Of these individuals, 32 males and 72 females were released into the eight glass vials, with each vial containing four males and nine females. Each new glass vial was provided with a new, previously frozen, lab-reared *M. cribraria* egg mass, to further evaluate rates of parasitism and suitability for rearing. The remaining adults were returned to the plexiglass growth chamber to be used as a maintenance colony.

### Morphological identification

Specimens were point-mounted and deposited in the Florida State Collection of Arthropods (FDACS-DPI, E2018-4411-1). Two specimens (one male and one female) were cleared in potassium hydroxide and slide-mounted in Canada balsam, according to a protocol modified from Noyes (1982). Whole-body images were taken with a Macropod® imaging system using a Canon EOS 6D Mark II camera, a EF 70-200mm lens and a 20X M Plan APO Mitutoyo objective lens. Image stacks were rendered using Helicon Focus® and edited in Adobe Photoshop®. Mandible images were taken on a Zeiss Axio Imager M2 Microscope with Axiocam 503 colour camera and Zen 2 software, using differential interference contrast lighting. Mandible image stacks were rendered using Zerene Stacker.

### DNA extraction, PCR and sequence assembly

Six *O. nezarae* individuals from the DPI colony were selected for DNA extraction and COI barcoding. Samples were collected directly into 70% EtOH and were air-dried two hours before proceeding to DNA extraction. DNA extractions of entire individuals were performed using DNeasy Blood and Tissue Kits (Qiagen). Extraction protocols followed the manufacturer’s recommendations with one minor adjustment: samples were not incubated for 10 minutes following tissue lysis and addition of Buffer AL. DNA extracts were quantified using a NanoDrop 2000 spectrophotometer (Thermo Scientific). At least 20 ng of template DNA was used per PCR.

The 5’-COI barcode region was PCR-amplified using the primers LEP-F1 and LEP-R1 (Hebert et al. 2004). PCRs were performed at 25 µl volumes using HiFi HotStart DNA Polymerase (Kapa Biosystems). PCR thermocycle conditions were: 1) initial denaturing at 95°C for 2:00 minutes followed by 32 cycles of steps 2–4, 2) 98°C for 20 seconds, 3) 40°C for 30 seconds, 4) 72°C for 30 seconds and 5) final extension at 72°C for 7:00 minutes. PCR products were verified by gel electrophoresis and cleaned for sequencing with QIAquick Gel Extraction Kits (Qiagen). Purified PCR products were Sanger sequenced in both directions using BigDye Terminator v3.1 (Applied Biosystems) chemistry on a SeqStudio Genetic Analyzer (Applied Biosystems). Sequence reads were trimmed and sequence contigs were assembled in Sequencher 5.4.6 (Gene Codes Corporation). COI barcodes generated during this study were deposited in GenBank (accessions MH996994-MH996999).

### Datamining, alignments and clustering analyses

To evaluate our new sequences in the context of the subfamily, all 5’-COI barcodes for *Ooencyrtus* and Encyrtinae were mined from the Barcode of Life Database (BOLD) (Ratnasingham and Hebert 2007). The tetracnemine species *Aenasius bambawalei* Hayat was selected as an outgroup based on the sister relationship between Tetracneminae and Encyrtinae recovered by Munro et al. (2011). The COI barcode dataset contained twenty *Ooencyrtus* sequences representing at least three identified species: *O. kuvanae* (Howard), *O. ferdowsii* Ebrahimi and Noyes and *O. nezarae* from Alabama, USA. The remaining nine *Ooencyrtus* sequences were determined only to the level of genus.

Sequences were aligned using ClustalW with default settings (Thompson et al. 1994) as implemented in MEGA7 (Kumar et al. 2016). The sequence alignment was trimmed on both ends to optimise for completeness of data coverage. Short COI sequences and sequences with many ambiguous bases were excluded from analysis. In total, the aligned COI barcode dataset was 562 bp and contained 204 encyrtine sequences with 99% data coverage (Table 1; Suppl. material 1). To visualise the clustering of the sequences in MEGA7, a neighbour-joining tree was inferred using distances computed by the Kimura 2-parameter model (K2P) with complete deletion of missing or ambiguous data (Kimura 1980, Kumar et al. 2016). Computing the K2P distances for the *Ooencyrtus* data was complicated by the lack of species-level identifications (i.e. sequences need to be grouped by the user to perform the calculations). To overcome this impediment, K2P distances for *Ooencyrtus* “species” were calculated using molecular operational taxonomic units (OTUs) recovered by species delimitation procedures.

### Phylogenetic tree searches and species delimitation analyses

The mPTP species delimitation programme requires a rooted, binary Newick tree for input (Kapli et al. 2017). Maximum likelihood analysis of the encyrtine COI matrix was conducted in W-IQ-TREE (Trifinopoulos et al. 2016). The matrix was partitioned by codon position. The best-fit model of sequence evolution for each partition was selected by ModelFinder (Kalyaanamoorthy et al. 2017), as implemented in W-IQ-TREE, using the Bayesian information criterion (TN+F+G4 first position; GTR+F+I+G4 second position; TIM+F+G4 third position). Bootstrap support values for the most likely tree were calculated using 10,000 ultrafast bootstrap replicates (Hoang et al. 2017). The bootstrap consensus tree was converted to Newick format in FigTree 1.4.0 (Rambaut 2012) for importation into the mPTP programme.

Delimitations computed in the mPTP programme used the minimum branch length threshold calculated with the “--minbr_auto” option. Heuristic maximum likelihood species delimitations were performed with a single coalescent rate averaged over all species. The confidence of the delimitations was assessed using the Markov Chain Monte Carlo (MCMC) sampling method. MCMC parameters included two independent runs with each run starting with the most likely delimitation, 100,000,000 MCMC iterations, and sampling of log-likelihoods every 10,000 MCMC iterations. The convergence of independent runs was evaluated by examining the sampled log-likelihood plots. The two runs converged within the first 1% of MCMC iterations.

Species delimitations were also produced using the server version of Automatic Barcode Gap Discovery (ABGD) (Puillandre et al. 2012). The results of ABGD depend heavily on parameters set by the user. We generally followed the ABGD methods of Pentinsaari et al. (2017), with the prior upper limit to intraspecific divergence (P) set to 0.01 for all tests. We also varied the relative gap width (X) from 0.1 to 2.0 at 0.1 intervals. The ABGD distance matrix was calculated using the K2P model with the transition/transversion ratio set to 1.05, as was calculated in MEGA7 from the alignment.

## Results

### Parasitism and emergence

The three previously frozen *M. cribraria* egg masses used to rear the first generation comprised 235 eggs. Of these, 65 were deemed unviable because they had desiccated prior to being frozen or during the freezing process, resulting in 170 available eggs. Of the available eggs, 122 individuals emerged: 36 males and 86 females. The second generation provided a total of 751 eggs, with 521 eggs deemed viable. Of the viable eggs, 345 were parasitised (66.2%) and 314 adult *O. nezarae* emerged: 119 males and 195 females. Rates of parasitism and emergence for each of the vials are provided in Table 2.

### Morphological identification

**Figure 1.**
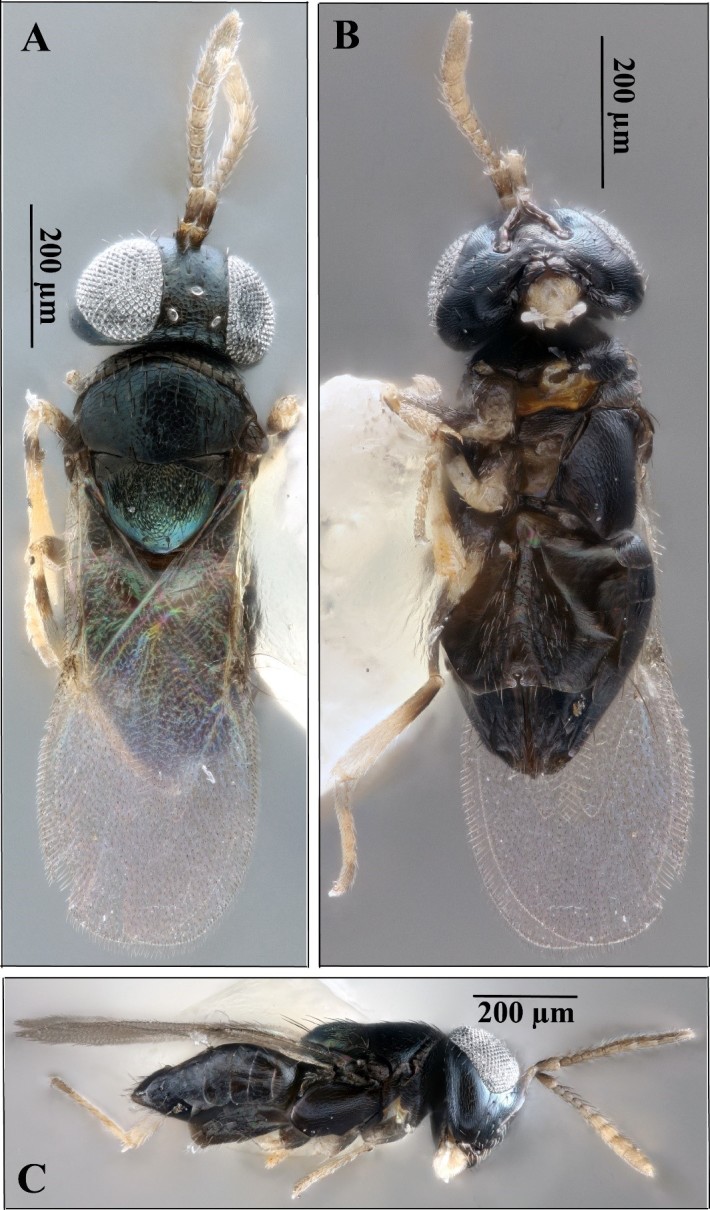
*Ooencyrtus nezarae*, female. **A.** Dorsal habitus; **B.** Ventral habitus; **C.** Lateral habitus.

**Figure 2.**
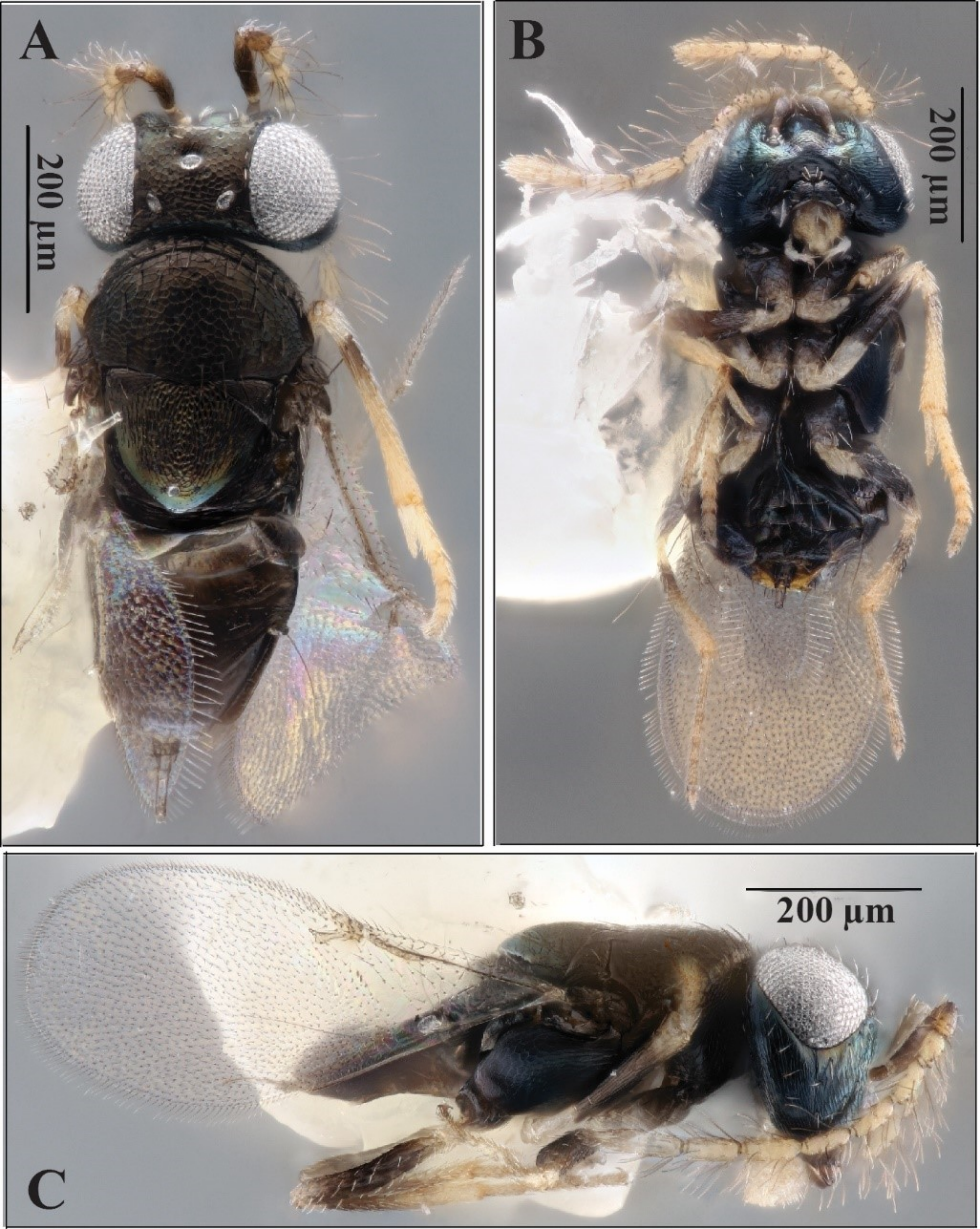
*Ooencyrtus nezarae*, male. **A.** Dorsal habitus; **B.** Ventral habitus; **C.** Lateral habitus.

**Figure 3.**
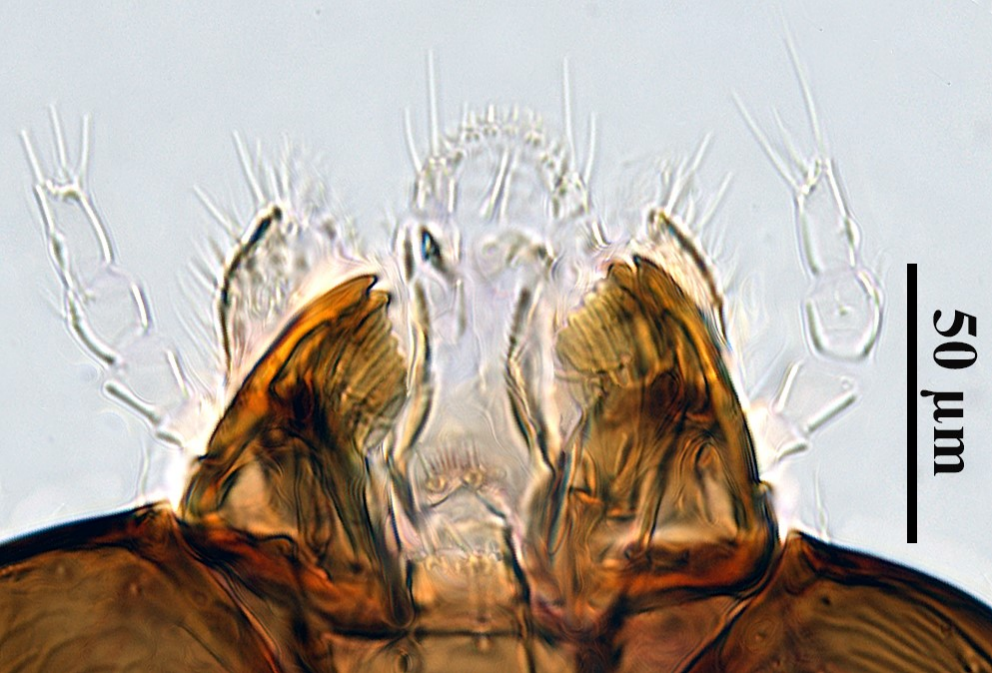
*Ooencyrtus nezarae* male mandibles.

Laboratory reared specimens were compared to descriptions in Ishii (1928), Zhang et al. (2005) and Ademokoya et al. (2018). The morphology of the specimens was consistent with the diagnoses given in these references (see Figs 1, 2, 3): frontovertex approximately 1/3 of head width; ocelli forming an obtuse triangle; posterior ocelli closer to compound eye than to occipital margin; mandible with crenulated truncation and distal tooth; stigmal vein of wings longer than postmarginal vein; linea calva open posteriorly; dark colouration on coxae, mesal femora, sub-basal tibiae and apical tarsi; the rest of the legs whitish-yellow; body dark with bluish metallic sheen; reticulation present on most of body; apex of scutellum smooth.

Ademokoya et al. (2018) noted that the Alabama population of *O. nezarae* exhibited differences in overall size and antennal morphometrics from the holotype. It was later confirmed via correspondence with J.S. Noyes at The Natural History Museum, London, that this discrepancy could be explained by allometric scaling of funicular segments (Ademokoya et al. 2018). The Alabama specimens were small (body length 0.70–0.77 mm) and the first two funicular segments of the female antenna were quadrate or slightly wider than long, in contrast to the descriptions of Ishii (1928) and Zhang et al. (2005), which described them as longer than wide. The female *O. nezarae* from Florida are approximately 0.8 mm in length and the first two funicular segments are roughly quadrate. These observations are consistent with the pattern of allometric scaling in these segments; i.e. larger specimens have proportionally longer funicular segments.

There is some disagreement between Ishii (1928) and Zhang et al. (2005) regarding the proportions of the third funicular segment in *O. nezarae*. Ishii (1928) describes its length as equal to its width, while Zhang et al. (2005) state that all funicular segments are longer than wide. In this respect, the Florida specimens more closely match the description given by Ishii (1928).

### Clustering analyses

**Figure 4.**
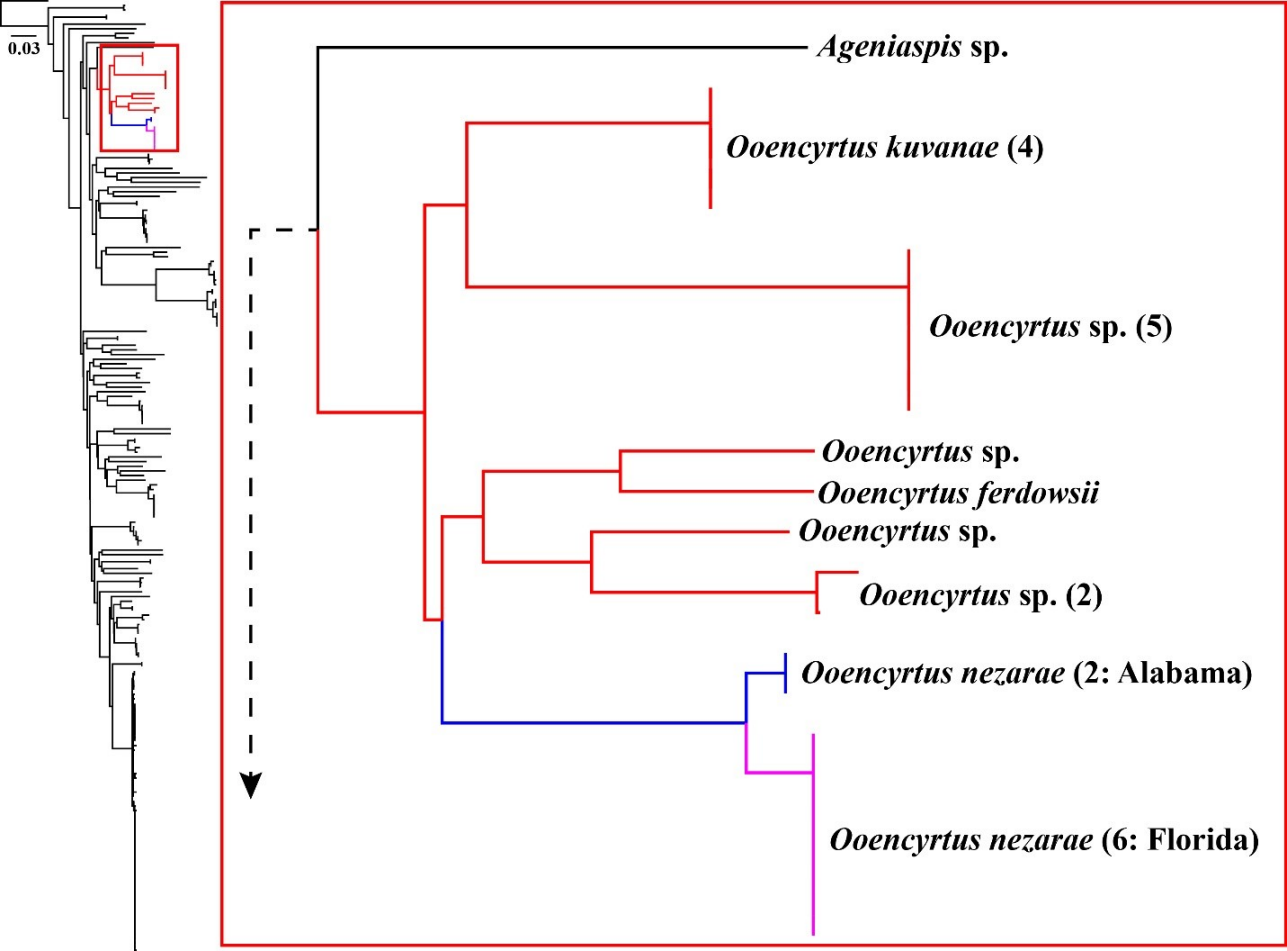
Kimura 2-parameter neighbour-joining tree showing the clustering of similar sequences in the Encyrtinae 5’-COI barcode dataset. Red branches indicate the *Ooencyrtus* cluster. Blue and fuchsia branches indicate *O. nezarae* from Alabama and Florida, respectively. Numbers in parentheses after the taxon name indicate how many sequences are represented in that cluster.

Neighbour-joining analysis excluded all positions with ambiguous data, leaving a total of 487 positions for inclusion (out of 562 possible positions) and recovered an *Ooencyrtus* group comprised of eight clusters (Fig. 4) (see Suppl. material 2 for groupings used for K2P distance calculations). Mean between-group K2P distances ranged from 5.4% to 13.7%. Mean within-group sequence divergences were low when comparisons were possible. The group containing sequences KF724500 and KF724501 had a mean within-group K2P distance of 0.8%. For *O. nezarae*, the Alabama and Florida samples had a mean between-group K2P distance of 1.3%. The sequences that form the two *O. nezarae* groups (Alabama and Florida) have zero within-group divergence. When taken together, the *O. nezarae* sequences ranged from 8.2% to 13.0% different from the other OTUs.

### Phylogenetic tree searches and species delimitation analyses

**Figure 5.**
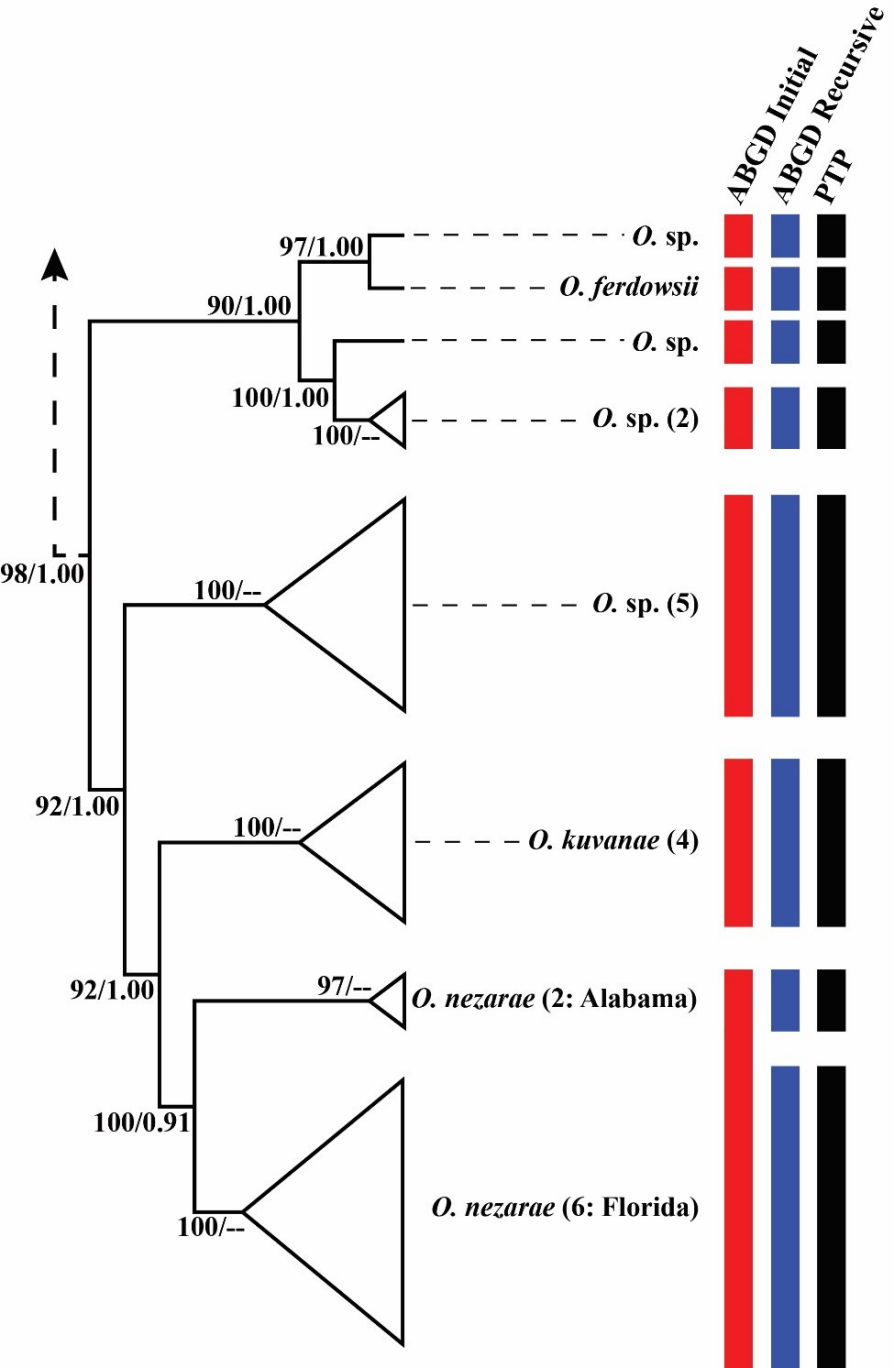
Transformed *Ooencyrtus* branch from the most likely tree for the Encyrtinae COI dataset. Each coloured bar represents an OTU delimited by the ABGD (initial and recursive partitions) and PTP procedures. Support values are ML bootstraps and the fraction of MCMC sampled delimitations in which a node was part of the speciation process in the PTP procedure, respectively. Numbers in parentheses after the taxon name indicate how many sequences are represented in that OTU.

W-IQ-TREE analyses found the most likely tree with a log likelihood score of -12138.342. ML bootstrap support for the recovered *Ooencyrtus* clade was strong (98%; Fig. 5). The internal nodes of the *Ooencyrtus* clade were also strongly supported, ranging from 92% to 100% ML bootstrap support (Fig. 5). Heuristic maximum likelihood species delimitations using PTP delimited 74 total species from the Encyrtinae COI barcode dataset and eight *Ooencyrtus* species (Fig. 5). This delimitation scheme supported the split of the Alabama and Florida *O. nezarae* into reciprocally monophyletic OTUs. Runs of the MCMC PTP analysis returned an average ML support of approximately 0.954 and an average standard deviation of support values amongst these runs of 6.9*10^-6^. Within the *Ooencyrtus* clade, the fraction of MCMC sampled delimitations, in which a node was part of the speciation process, was 1.00 in all cases except for *O. nezarae*, which was 0.91 (Fig. 5). This indicates that the delimitation that split Alabama and Florida *O. nezarae* was not always recovered by this analysis.

In ABGD, all tested minimum relative gap widths returned initial delimitations of 68 species from the Encyrtinae dataset at P = 0.01. In this same range of minimum relative gap widths, ABGD returned recursive delimitations of 71 or 72 from the encyrtine dataset at P = 0.01. Across all tests, the initial delimitations recognised seven *Ooencyrtus* species and lumped *O. nezarae* from Alabama and Florida into a single OTU (Fig. 5). Across all tests, the recursive delimitations recognised eight *Ooencyrtus* species (matching the PTP delimitation) and split *O. nezarae* from Alabama and Florida into two OTUs (Fig. 5).

## Discussion

Our testing indicates that the Florida *O. nezarae* COI sequences are 1.3% different from the Alabama *O. nezarae* sequences. This was the largest infraspecific sequence divergence observed amongst the *Ooencyrtus* species in this study. PTP and ABGD recursive delimitations separated the Florida *O. nezarae* into distinct OTUs due to this degree of sequence divergence. Based on morphological data indicating that the Alabama and Florida *O. nezarae* are conspecific, inconsistency between molecular species delimitation methods and lack of statistical support for the PTP delimitation, we consider the divergent Florida *O. nezarae* COI sequences to represent a distinct haplotype rather than evidence of a separate species. *Ooencyrtus nezarae* is a widely distributed species occurring in China, Japan, Thailand, South Korea, Brazil and now the south-eastern United States (Kobayashi and Cosenza 1987, Zhang et al. 2005, Ademokoya et al. 2018, Noyes 2018). COI sequence data is only available from the adventive populations of *O. nezarae*, highlighting the need for more data from across the native range of this species. Additional COI barcodes from the native range of *O. nezarae* could help establish whether the population present in Florida is from the same, yet genetically diverse, introduction as the Alabama population or represents a separate introduction event.

Implications for Florida agriculture merit further investigation. *Ooencyrtus nezarae* has a wide host range, including at least four hemipteran families (Noyes 2018). It remains unknown whether its arrival will impact other pentatomoid species of agricultural or ecological importance. Its distribution through the region should be monitored, as it has potential to move south through Miami, the Keys and the Caribbean.

Future research may investigate the performance of *O. nezarae* on other pentatomoid host species of interest to Florida agriculture. Morphometrics of parasitoids reared from different sizes and species of eggs are expected to reveal allometric scaling. Due to this, antennal morphometrics are not recommended as diagnostic characters for this species, although they may have some utility in determining the host origin of specimens.

Competition between egg parasitoid species has the potential to impact populations of introduced and native biological control agents. For example, egg parasitoids, such as *Trissolcus* species, are known to exhibit intra-guild competition, both in the form of exploitative competition when parasitoids of another genus are present and in the form of interference competition when multiple *Trissolcus* species are present (Sujii et al. 2002). Observations and preliminary results suggest that *O. nezarae* is more prolific than *Paratelenomus saccharalis* (Ademokoya et al. 2018). Consequently, further research is required to assess effects on parasitoid-host interactions in *M. cribraria*, as well as on other systems involving native egg parasitoid and pentatomoid species.

At the time of writing, fifteen generations of *O. nezarae* have been reared successfully in captivity using previously frozen, lab-reared *Megacopta cribraria* eggs, collected 6 – 14 months prior to use. This is the first recorded instance of *O. nezarae* being reared in captivity using previously frozen eggs or from eggs kept in storage, refrigerated or frozen, for more than 200 days (Alim and Lim 2010). They have, thus far, had no issues with pathogens or any noticeable loss of reproductive vigour. The Florida laboratory colony exhibited lower parasitism rates (41% – 78% compared to 82% – 100%) than field-collected material from Alabama. Sex ratios were similarly female-biased (Ademokoya et al. 2018). It is important to note that laboratory data may not represent sex ratio and parasitism rate in the field, especially when the captive colony has been reared from frozen eggs.

## Conclusions

*Ooencyrtus nezarae* is an interesting new arrival to Florida; one that merits further study. Through rearing, we have observed that it is a hardy, generalist egg parasitoid well-adapted to a subtropical climate. The ease by which it can be reared suggests it may be a suitable candidate for future biological control projects requiring a generalist parasitoid, such as augmentative biological control programmes for growers dealing with hemipteran pests.

Despite possible beneficial applications this insect may have, however, the fact remains that it is not indigenous to the United States. Additional research is necessary to determine if *O. nezarae* may negatively impact populations of native egg parasitoids or beneficial hemipterans. Additionally, native populations should be sampled and sequenced to elucidate why the Florida population is of a different haplotype than the previously-documented Alabama population. Determining where these respective non-indigenous populations originated may provide novel information regarding the pathways by which invasive organisms arrive in the south-eastern United States.

## Supplementary Material

6C27CAFC-7800-56F9-A147-56BF37A09491 10.3897/BDJ.8.e36440.suppl1 Supplementary material 1 COI Barcode Dataset Data type: Genomic
File: oo_303610.fas https://binary.pensoft.net/file/303610 M. R. Moore

57D1F505-14B5-5089-B8E5-A132A5A39095 10.3897/BDJ.8.e36440.suppl2 Supplementary material 2 K2P Distance Groupings Data type: K2P distance groupings
File: oo_303828.txt https://binary.pensoft.net/file/303828 M. R. Moore

## Figures and Tables

**Table 1.** COI GenBank Accessions and BOLD sequence IDs utilised in this study.

**Taxon**	**Accession Number or BOLD Sequence ID**
*Aenasius bambawalei*	KY837980
*Ageniaspis citricola*	KF850108
*Ageniaspis* sp.	KF850121
*Avetianella longoi*	JQ688066-JQ688068
*Bothriothorax* sp.	KR894744
*Comperiella bifasciata*	JQ268910-JQ268911
*Copidosoma agrotis*	KF850094
*Copidosoma aretas*	KF850130
*Copidosoma boucheanum*	KF850099
*Copidosoma cervius*	KF850097
*Copidosoma chalconotum*	KF850101
*Copidosoma coimbatorense*	KF850107, KF850131
*Copidosoma floridanum*	ASEN036-07, ASEN039-07, ASHYB1605-09, BCHYM1992-14, GMHDP240-13, KF850096, KF850136, KF850141, KF850144, KM996223, KR406982, KR414572, KR420962, KR782588, KR784194, KR786198, KR787723, KR787833, KR788049, KR789152, KR789176, KR789450, KR789626, KR789844, KR790973, KR791769, KR792534, KR793914, KR794229, KR794305, KR795395, KR799312, KR800638, KR803393, KR804110, KR804647, KR805138, KR805502, KR806827, KR807027, KR808577, KR876352, KR876834, KR877346, KR881873, KR882690, KR887258, KR890279, KR890965, KR891435, KR893952, KR897932, KR900845, KR902174, KT609386, KT610656, MF898745, MG440639, MG443669, MG444029, MG444896, MG445072, MG445123, MG445159, OPPFQ3505-17, OPPFS2202-17, OPPQE2701-17
*Copidosoma fuscisquama*	KF850110
*Copidosoma koehleri*	KX443094, KX443096
*Copidosoma lucidum*	KF850129
*Copidosoma noyesi*	KF850105
Copidosoma nr. notatum	KF850117
Copidosoma nr. noyesi	KF850124, KF850128
Copidosoma nr. peticus	KF850125-KF850126, KF850133
Copidosoma nr. subalbicorne	KF850109, KF850112, KF850137-KF850138
Copidosoma nr. thebe	KF850134-KF850135
*Copidosoma peticus*	KF850098
*Copidosoma phaloniae*	KF850100
*Copidosoma primulum*	KF850102, KY831867, KY831980, KY837727, KY840018, KY844984
*Copidosoma serricorne*	KF850116
*Copidosoma sosares*	KF850113
*Copidosoma* sp.	BARSB322-16, BARSI478-16, BARSJ296-16, BARSL101-16, KR407823, KR783521, KR788209, KR793794, KR797140, KR803377, KR803644, KR803783, KR800219, KR804761, KR806647, KR808671, KR874151, KR877046, KR878229, KR884641, KR886957, KR897641, KR897987, KR899311, KR901314, KX535016, MF898523, MF902116, MF906707, MF907230, MG444508
*Copidosoma thebe*	KF850114
*Copidosoma transversum*	KF850120
*Copidosoma truncatellum*	KF850093
*Copidosoma varicorne*	
*Copidosomopsis meridionalis*	KF850103
*Copidosomopsis nacoleiae*	KF850118
Encyrtinae sp.	MG444525
*Metaphycus flavus*	FM210164, GMESJ755-14
*Metaphycus groenlandicus*	KR406522, KR783093, KR784386, KR874706, KR890545, KU373887, MG444018, OPPFC1281-17
*Metaphycus* sp.	BCHYM1954-14
*Neastymachus axillaris*	KM095502
*Ooencyrtus ferdowsii*	KR270994
*Ooencyrtus kuvanae*	KX868565, KX868567-KX868569
*Ooencyrtus nezarae*	KY964494-KY964495, MH996994-MH996999
*Ooencyrtus* sp.	KC149976, KF724500-KF724501, KF724504, KF850146, KF888626-KF888629
*Paralitomastix* sp. Noyes03	ASHYF2008-11, HM373219
*Pseudencyrtus* n. sp.	KU373908
*Pseudencyrtus* sp.	MG442888
*Psyllaephagus pilosus*	NZHYM109-10
*Syrphophagus aphidivorus*	JF906507, JX507455, KF597765-KF597773, KF894410
*Syrphophagus* sp.	KF597774-KF597779

**Table 2.** Rates of parasitism and emergence in the second generation *Ooencyrtus nezarae*.

**Vial**	**Available Host Eggs**	**Number Parasitised**	**Number Emerged**	**Rate of Parasitism**	**Proportion Female**
**1**	94	59	52	62.8%	0.62
**2**	78	55	54	70.5%	0.61
**3**	78	50	37	64.1%	0.43
**4**	69	54	52	78.3%	0.64
**5**	41	26	22	63.4%	0.64
**6**	34	14	13	41.2%	0.69
**7**	66	43	42	65.2%	0.69
**8**	61	44	42	72.1%	0.67
**Total**	521	345	314	66.2%	0.62
